# Changes in muscle coordination patterns induced by exposure to a viscous force field

**DOI:** 10.1186/s12984-016-0164-3

**Published:** 2016-06-16

**Authors:** Fabio Oscari, Christian Finetto, Steve A. Kautz, Giulio Rosati

**Affiliations:** Dept. of Management and Engineering, University of Padua, Via Venezia 1, Padua, 35135 Italy; Dept. of Health Sciences and Research, Medical University of South Carolina, 77 President Street, MSC 700, Charleston, SC 29425 USA; Ralph H. Johnson VA Medical Center, Charleston, SC 29425 USA

**Keywords:** Motor adaptation, Learning, Motor modules, Viscous field, Dynamic perturbation, Robotics, Haptics

## Abstract

**Background:**

Robotic neurorehabilitation aims at promoting the recovery of lost function after neurological injury by leveraging strategies of motor learning. One important aspect of the rehabilitation process is the improvement of muscle coordination patterns, which can be drastically altered after stroke. However, it is not fully understood if and how robotic therapy can address these deficits. The aim of our study was to find how muscle coordination, analyzed from the perspective of motor modules, could change during motor adaptation to a dynamic environment generated by a haptic interface.

**Methods:**

In our experiment we employed the traditional paradigm of exposure to a viscous force field to subjects that grasped the handle of an actuated joystick during a reaching movement (participants moved directly forward and back by 30 *cm*). EMG signals of ten muscles of the tested arm were recorded. We extracted motor modules from the pooled EMG data of all subjects and analyzed the muscle coordination patterns.

**Results:**

We found that the participants reacted by using a coordination strategy that could be explained by a change in the activation of motor modules used during free motion and by two complementary modules. These complementary modules aggregated changes in muscle coordination, and evolved throughout the experiment eventually maintaining a comparable structure until the late phase of re-adaptation.

**Conclusions:**

This result suggests that motor adaptation induced by the interaction with a robotic device can lead to changes in the muscle coordination patterns of the subject.

## Background

Human subjects can adapt to a novel dynamic environment during reaching movements [1]. When subjects are abruptly exposed to the novel environment (dynamic perturbation), large trajectory errors (direct effect) are shown, compared with unperturbed movements (baseline). However, with practice, hand trajectories during the exposure to the novel perturbation gradually converge to a path very similar to that observed at baseline. The recovery of performance within the changed mechanical environment is called motor adaptation [1–4]. The proof that motor adaptation happens is the presence of after effects as response to the sudden removal of such dynamic perturbation after the training phase. After effects manifest as trajectory errors comparable but opposite to direct effects, which gradually decrease to baseline levels after further unperturbed training(re-adaptation). This behavior is commonly recognized as the human ability to form an internal model of the environment that the central nervous system (CNS) uses to predict and compensate for the forces imposed by the environment [1–4]. From a neuromuscular point of view, evidences suggest that the generation of the new internal model could manifest as gradual changes in the timing and amplitude of muscle activation [5]. However, it is still unknown how this information is translated into the specific pattern of muscle activation selected to generate the desired output [2–4, 6]. In fact, the same force could be obtained with a multitude of different muscle activation patterns. Adaptation to the novel environment could be obtained by independently adjusting the activity of all participating muscles, which, due to the redundancy of the musculoskeletal system, would lead to an infinite number of solutions generating the same output. Alternatively, the same goal could be obtained by conserving most of the muscle coordination used during unperturbed motion.

Recent findings suggest that the CNS could adopt a modular organization to coordinate the muscles participating in a functional movement or in an isometric task [7–13]. According to this theory, muscles could be grouped into motor modules, where each module unites muscles that activate synchronously [7, 14–17]. Motor modules could therefore explain the muscle coordination strategies used during a specific task. This makes the analysis of motor modules during motor adaptation particularly interesting, as it can give an insight into how the learning of a novel environment affects muscle coordination.

Changes in muscle coordination patterns during motor adaptation have been rarely investigated in the literature. Berger et al. [18] analyzed the effect of virtual surgeries in an EMG-driven isometric force generation task. A virtual surgery consisted in changing the mapping between the activation of a muscle and the resulting end-effector force. The authors found that subjects adapted to the perturbation of the muscle to force mapping slower when it was incompatible with their motor modules, i.e. when the activity of muscles that were part of a module generated a resulting force equal to zero. This suggests that motor adaptation is more challenging when subjects need to learn new coordination strategies. Gentner et al. [6] studied the robustness of motor modules during an isometric force generation task in the presence of a visuomotor perturbation. The perturbation was generated by changing the mapping between the recorded force and the resulting acceleration of a virtual object. The authors found that the structure of motor modules was stable across different mappings, and that subjects adapted to the visuomotor perturbation by changing the recruitment pattern of the modules. Both these studies suggest that motor adaptation is achieved by preferentially recruiting the same modules used during an unperturbed condition. However, no study so far has analyzed motor modules during adaptation to a dynamic environment.

In our experiment the traditional paradigm of the exposure to a viscous force field was applied to subjects grasping the handle of an actuated joystick during reaching movements. Throughout the experiment, we recorded the EMG signals from ten muscles of the tested arm. Muscle coordination patterns were analyzed by extracting motor modules from the recorded EMG signals. The aim of our experiment was to analyze the task-specific modules of the tested movement to find whether muscle coordination patterns changed during and after motor adaptation to a dynamic perturbation. Our first hypothesis was that motor modules extracted from the unperturbed motion (baseline) could also explain the muscle activity in the later phases of the experiment, requiring an adjustment of their activation in order to cope with the external perturbation. Secondly, we hypothesized that the adaptation to the force field would lead subjects to change their muscle coordination by adopting previously unused coordination strategies that could be summarized as complementary modules, and which would be retained even after the removal of the perturbation.

## Methods

### Subjects

Twelve healthy subjects (mean age 26.2 ± 1.4 years, 6 male, 6 female) participated in the experiment. All participants were right-handed; they reported normal vision, no color blindness, no hearing problems, and no orthopedic condition affecting arm mobility. Written informed consent for participation in the experiments and for the publication of this report was obtained from all the subjects. The experiment received the ethical approval of the Scientific Commission of the University of Padua.

### Setup

**Fig. 1:**
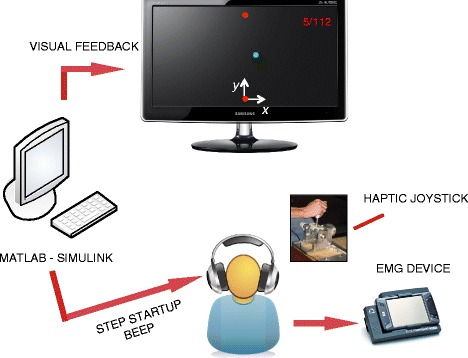
Experimental setup and feedback system. Subjects sat on a chair in front of an LCD screen, grasping a 2-degrees-of-freedom semi-active joystick with their right hand. The direction of motion (*y*) was always parallel to the subject’s sagittal plane. During the experiment, EMG data of 10 surface muscles of the right upper limb were recorded. The subjects received video, audio, and force feedback

The experimental setup is shown in Fig. 1. Subjects sat on a chair in front of an LCD screen, grasping a 2-degrees-of-freedom (DoF) semi-active joystick with their right hand. The direction of motion (*y*) was always parallel to the subject’s sagittal plane and was coincident with the joystick’s passive DoF, while the active DoF was oriented along the mediolateral direction (*x*). The joystick was a partially re-designed version of that presented in [19], and was modified to extend the movement range to 30 cm and to increase the maximum force applied to the subject’s hand to 5 N. These changes were required to elicit more distinct and repeatable muscle activation patterns, and to increase the muscular activity when a force field was provided. The joystick was controlled by means of a Sensoray 626 data acquisition board and a Simulink model running in real time at a 200 Hz update rate. The graphical interface displayed on the LCD screen was implemented in DirectX and was synchronized with the Simulink model. In addition to visual feedback, subjects were provided with auditory cues signaling the start of each movement. The details of the graphical and audio cues are described in the next section.

During the experiment, we recorded electromyographic (EMG) data of 10 surface muscles of the right upper limb. We used disposable Ag/AgCl electrodes with 26 mm spacing and a 16 channel electromyograph (BTS Engineering PocketEMG). Electrodes were placed above the following muscles adopting SENIAM recommendations [20]: anterior, medial, and posterior deltoid (*AD*, *MD*, *PD*), infraspinatus (*IS*), trapezius (upper fibers, *TP*), pectoralis major (*PM*), biceps brachii (long head, *BI*), triceps brachii (lateral head, *TR*), extensor carpi ulnaris (*EC*) and flexor carpi radialis (*FC*). EMG data was sampled at 2 kHz and synchronized with the Simulink model.

### Experimental protocol

Subjects were asked to perform forward (+*y* direction) and backward (−*y* direction) 30 cm-long reaching movements between two fixed targets, as straight as possible. We chose this task, used in previous experiments on motor adaptation (e.g., [21–23]), because it is simple and its repetitive nature is well suited for the analysis of motor modules. The *task* consisted in 112 movements (*steps*), corresponding to 56 forward (*reaches*) and 56 backward (*returns*) movements. A red circular cursor on the LCD screen indicated the position of the current target. The target’s diameter was about 1 cm both in the joystick space and on the screen. A green circular cursor of the same size was printed on the screen, whose *x* and *y* position corresponded to the location of the joystick’s handle. Subjects were instructed to start each step only after receiving an auditory cue, and then to move as fast as possible towards the target. The signal tones were equally spaced at 1.8 s and were used to standardize the time between two steps among subjects. Subjects were allowed a 30 s warm-up to practice the “rhythm” dictated by the audio cue.

Before beginning the experiment, chair height, joystick position and arm posture were adapted to standardized working conditions. With the joystick handle positioned at the center of the workspace, the position and height of the chair were adjusted so that the subject’s shoulder was abducted 45°, the elbow was flexed at 90° and the forearm was horizontal and parallel to the sagittal plane. Reaches started 15 cm behind this position, with the shoulder still abducted 45° and slightly extended, and with the elbow flexed beyond 90°. At the end of each forward movement, the shoulder was slightly flexed and the elbow was flexed less than 90°. Conversely, returns implied movements of shoulder extension and elbow flexion since start and end positions were inverted.

The experiment started with a sequence of 14 unperturbed steps, with the joystick completely passive. At the 15th step, a dynamic perturbation was introduced and lasted until the 56th step, after which it was removed. The perturbation consisted of a viscous force field *f*_*x*_, generated by the joystick. The lateral force was computed as a function of the velocity along the motion axis (*y*):

1 $$  \mathbf{f} = \left\{ \begin{array}{c} f_{x} \\ f_{y} \end{array} \right\} = \left[ \begin{array}{cc} b_{1,1} & b_{1,2} \\ b_{2,1} & b_{2,2} \end{array} \right] \cdot \left\{ \begin{array}{c} v_{x} \\ v_{y} \end{array} \right\}  $$

where all the elements of the viscosity matrix were set to zero except for *b*_1,2_=10 Ns/m. The endpoint force **f** is given in Newtons, the velocity **v** in meters per second.

After 98 movements, the experiment was paused and subjects rested their hand on a table. Following a 5 min rest period, subjects executed an additional 14 movements in unperturbed condition. These final steps were used to test the retention of muscle coordination patterns in absence of the force field and after a pause.

We grouped the movements of the task into 10 main *phases*, according to the motor adaptation literature [1, 21–24]:

1. *Baseline*: the initial movements performed without perturbation (steps 1–14).
2. *Direct effect*: the first cycle (reach + return) with exposure to the perturbation (steps 15–16).
3. *Adaptation (Early)*: the following 12 reaches, during which the subjects started to adapt to the perturbation (steps 17–28).
4. *Adaptation (Medium)*: the central phase of adaptation (steps 29–42).
5. *Adaptation (Late)*: the last 14 reaches before removing the perturbation (steps 43–56).
6. *After effect*: the first cycle (reach + return) after removing the perturbation (steps 57–58).
7. *Re-Adaptation (Early)*: the phase during which the subjects started to re-adapt to movements without perturbation (steps 59–70).
8. *Re-Adaptation (Medium)*: the second phase of re-adaptation (steps 71–84).
9. *Re-Adaptation (Late)*: the last phase of re-adaptation (steps 85–98).
10. *Follow-up*: after a pause of 5 min, other 14 movements were performed without perturbation (steps 99–112).

### Extraction of motor modules

Motor modules were extracted from the recorded EMG data using a custom MatLab script. The EMG signals were high-pass filtered at 30 Hz (4th order zero-lag Butterworth filter) in order to remove heart beat artifacts [25, 26], demeaned, full-wave rectified and low-pass filtered at 4 Hz (4th order zero-lag Butterworth filter). Each EMG channel was normalized by its maximum average value over a window of 90 ms moving over the whole dataset, calculated from the demeaned and rectified signal. Since all recorded muscles participated in the movement, this normalization procedure allowed us to detect the task-specific peak activity. We then identified the time frame during which the subject was moving the joystick, and used only the EMG data recorded during the motion for further analysis. Movement onset was defined as the instant when the joystick velocity along *y* increased above 5 % of its maximum value, and movement end as the instant when the velocity decreased below the same 5 % threshold. Subsequently the EMG data of each step was subsampled into 100 equally spaced samples, which allowed us to reduce the computational effort of the remaining analyses and guaranteed that every step was given the same weight during the extraction of motor modules.

We approximated the recorded EMG data (**V**) as a linear combination of *n*_*m*_ motor modules (**H**), multiplied by time varying activation coefficients (**W**). Each module stands for a time-invariant balance of activation between the recorded muscles, represented as a 1×10 vector of normalized muscle weights, and is scaled in time by its activation coefficient [7, 14, 17, 27, 28]. This approximation can be formalized as follows:

2 $$ \mathbf{V} \cong \mathbf{W} \mathbf{H}   $$

Where **V** is a matrix containing the EMG signals (one row per sample, one column per channel), **W** represents the activation coefficients (one row per sample, *n*_*m*_ columns), and **H** is a matrix with one motor module on each row.

We solved Eq. (2) by performing a non-negative matrix factorization (NNMF) using a novel variant of the Alternating Least Squares (ALS) algorithm [29, 30]. Instead of extracting motor modules independently from the data of each subject, or from the pooled data set of all subjects, we adopted a hybrid approach which obtained modules from the pooled data set while allowing for limited individual variability in the muscle weights. This approach leverages the results of previous studies, showing that modules tend to be similar across subjects [9, 17], to increase the size of the data set processed by the algorithm and to reduce the effect of noise on the modules’ structure. Furthermore, it allows for small individual differences in the muscle weights to better capture each subject’s muscle activation patterns. The main difference to a traditional NNMF-ALS algorithm is that at each iteration the module weights are calculated in two steps. First, the pooled data of all subjects is used to calculate modules that approximate the whole data set. Second, modules are extracted individually for each subject, but muscle weights are constrained to be within ±40 *%* of the overall modules. The 40 % threshold was selected as it leaves sufficient room for individual variability, while preliminary data suggested that it keeps the extracted modules comparable across subjects (dot products of at least 0.8 between modules of different subjects).

We extracted motor modules in two stages. In the first stage, we analyzed the unperturbed muscle coordination patterns by extracting modules only from the baseline EMG signals. The factorization was run multiple times with a different constraint on the number of motor modules, starting at *n*_*m*_=1 and ending at *n*_*m*_=10. We assessed the approximation error of the factorization by calculating the variability of the EMG data accounted for [28, 31]:

3 $$ VAF = 1 - \frac{\|\mathbf{V}-\mathbf{W} \mathbf{H}\|_{F}^{2}}{\|\mathbf{V}\|_{F}^{2}}   $$

We calculated the VAF for each subject and for each single step. The number of modules required to describe the EMG data was determined by requiring a minimum VAF of 95 *%* for each subject, and of 80 *%* for each step in order to guarantee an excellent overall reconstruction, and at the same time a sufficient detail on a single step [32, 33].

Before looking at changes in muscle coordination following adaptation to the perturbation, we verified that the extracted baseline modules were stable during the unperturbed condition. For this purpose, we extracted modules from only the first half of the baseline (first 8 steps), and used them to reconstruct the second half of the baseline (last 6 steps). The baseline modules where considered to be stable if the reconstruction of the second half of the baseline resulted in *VAF*≥95 *%* for each subject.

In the second stage, we used the previously obtained baseline modules, in combination with newly calculated complementary modules, to reconstruct the EMG data of the following phases. We allowed for a variable number of complementary modules, which were calculated independently for each phase. This approach allowed us to determine how well the baseline modules explained the muscle activity in the successive phases, and to explore any additional coordination strategies needed during and after the exposure to the force field. The advantage of this method is that changes in muscle activation patterns are detected in two ways: the adjustment of the activation of the baseline modules and the aggregation of any changes in muscle coordination into complementary modules. This hybrid factorization was obtained by slightly modifying the NNMF algorithm in order to constrain the first rows of **H** to be equal to the baseline modules:

4 $$ \mathbf{H} = \left[ \begin{array}{l} \mathbf{H}_{baseline} \\ \mathbf{H}_{complementary} \end{array} \right]   $$

We repeated the factorization with an increasing number of complementary modules, starting at zero until the total number of rows of **H** was equal to 10. The number of complementary modules needed to explain the muscle activation patterns was determined by requiring the same level of VAF as in the first stage (95 *%* for each subject, 80 *%* for each step). The factorization was performed using the same two step process used for baseline and allowing the same ±40 *%* variability in the weights of the complementary modules.

### Data analysis

The first step in the data analysis was to assess whether motor adaptation had taken place during the execution of the experiment. A commonly used indicator for motor adaptation is the kinematic deviation from the desired trajectory, which has been shown to tend towards the baseline value as the subject adapts to the perturbation [1]. The left–right *average weighted position error* [21, 23] was used as a measure of kinematic performance and was calculated for every subject, every phase and every movement direction (reach and return):

5 $$  e^{s}_{d,p} = \frac{1}{M_{d,p}} \sum_{h=1}^{M_{d,p}} \left(\sum_{i=1}^{N_{h}} \frac{-\text{sign}{\left(v_{y_{i}} \right)} \cdot \left(x_{i}/\left|{Y_{t}}\right|\right)}{N_{h}} \right)  $$

where *s* indicates the subject, *d* denotes the movement direction and *p*=1÷10 the phase; *M*_*d,p*_ is the number of steps in phase *p*; *x*_*i*_ and *v*_*yi*_ are, respectively, the current *x* position and *y* velocity of the hand (*i*−*th* sample); *Y*_*t*_=150 mm is the *y* position of the target to normalize the error; *N*_*h*_ is the number of samples in step *h*.

In order to better analyze the motion kinematics, we also looked at changes in the end point trajectory. For this purpose, we used the velocity profiles along the *y* axis of each movement to calculate two additional metrics.

To catch differences in the shape of the motion profiles, we calculated the *average correlation coefficient* of the velocity profiles of each phase compared to the mean velocity profile at baseline. This metric was calculated for every subject, every phase and every movement direction:

6 $$  c^{s}_{d,p} = \frac{1}{M_{d,p}} \sum_{h=1}^{M_{d,p}} \text{corr}\left(v_{y_{h}}, V_{y_{b}} \right)  $$

where $v_{y_{h}}$ is the *y* velocity profile in step *h* and $V_{y_{b}}$ is the mean velocity profile along *y* axis in baseline for the subject *s* in the phase *p* and depending on direction *d*; corr computes Pearson’s linear correlation coefficient.

Moreover, we calculated the *average travel time*, defined as the time necessary to move the hand from the start position to the target position, to capture temporal differences in the motion profiles. This metric was calculated for every subject, every phase and every movement direction as follows:

7 $$  t^{s}_{d,p} = \frac{1}{M_{d,p}} \sum_{h=1}^{M_{d,p}} \frac{t_{h}}{T}  $$

where *t*_*h*_ is the time travel in step *h*, normalized with respect to the time available between two steps, *T*=1.8 s.

The value of each metric for the whole cycle ${e^{s}_{p}}$, ${c^{s}_{p}}$, ${t^{s}_{p}}$ was obtained by averaging, respectively, the values of $e^{s}_{d,p}$, $c^{s}_{d,p}$, $t^{s}_{d,p}$ over reach and return.

Motor adaptation from the perspective of motor modules was investigated by analyzing how the activity of the baseline modules evolved during the exercise, and how the number and structure of the complementary modules changed during and after the exposure to the force field. In order to detect changes in module activity, we calculated the average activation of each module in each phase. The *average module activation* was calculated independently for each subject and separately for reach and return movements:

8 $$  w^{s}_{d,p,m} = \frac{1}{M_{d,p}} \sum_{h=1}^{M_{d,p}} w_{m,h}  $$

where, in addition to the notation of (5), *m*=1÷*n*_*m*_ denotes the module while *w*_*m,h*_ is the average activation value of module *m* during step *h*. By calculating the mean of the module activation in the two movement directions, we also obtained the value for a whole cycle ($w^{s}_{p,m}$).

We then compared the complementary modules to evaluate whether they were consistent across different phases of the experiment, or if different phases required different coordination strategies. This was done by applying a k-means cluster analysis to the pooled set of complementary modules of all phases. We used the scalar product as the distance measure and repeated the clustering 200 times starting from a random initial guess. The number of clusters was determined by using the smallest number for which the elements of each cluster had a scalar product of at least 0.9 with the respective centroid. Each cluster was regarded as a muscle coordination strategy specific to one or more phases.

Since the complementary modules are likely to represent coordination strategies aimed at balancing the external force, we were also interested in analyzing their timing with respect to the perturbation. We calculated the difference in time between the peak activity of each complementary module and the velocity peak, to reveal whether a module anticipated the perturbation, was synchronous with the perturbation or was delayed with respect to the perturbation. This metric was normalized by the movement time, and was averaged over each phase:

9 $$  \Delta t^{s}_{d,p,m} = \frac{1}{M_{d,p}} \sum_{h=1}^{M_{d,p}} \frac{t_{wmax,h} - t_{vmax,h}}{t_{h}}  $$

where, *t*_*wmax,h*_ is the time of peak activity of module *m* at step *h*, *t*_*vmax,h*_ is the time of peak velocity at step *h* and *t*_*h*_ is the movement time at step *h*.

Normality tests (Shapiro-Wilk normality test and D’Agostino-Pearson omnibus normality test) indicated a Gaussian distribution of all metrics defined.

As statistical tools, we ran a two-way within-subjects ANOVA for all the kinematic metrics ($e^{s}_{d,p}$, $c^{s}_{d,p}$, $t^{s}_{d,p}$), with phase and direction as within factors, to test the kinematic performance across phases and directions. Instead, a three-way within-subjects ANOVA for $w^{s}_{{p,m,d}}$ and $\Delta t^{s}_{{p,m,d}}$, with phase, module, and direction as within factors, was carried out in order to find differences of each module activation and the time synchronization of its peak with the velocity peak due to phase or direction. When a module was not present in all phases, we performed a separated two-way ANOVA for that module with phase and direction as within factor.

In the presence of significant effects, pair wise post-hoc comparisons (Tukey’s test) were performed, reporting here the interesting ones only. When a module was present in a phase only a pairwise t-test was used to compare between directions.

Additionally, to help in the biomechanical interpretation of all baseline modules, we looked at changes in the modules’ activity the initial and terminal part of each movement. This was done through pairwise t-tests, comparing the average module activation during the first 20 *%* of each movement to that during the last 20 *%*.

One participant exhibited large variable errors and when questioned after the experiment it was evident that the description of the task had been misunderstood. Also, during the exercise of another participant we encountered a system malfunction resulting in data loss. Thus, the data of these two subjects was excluded.

## Results

### Kinematic performance

**Fig. 2:**
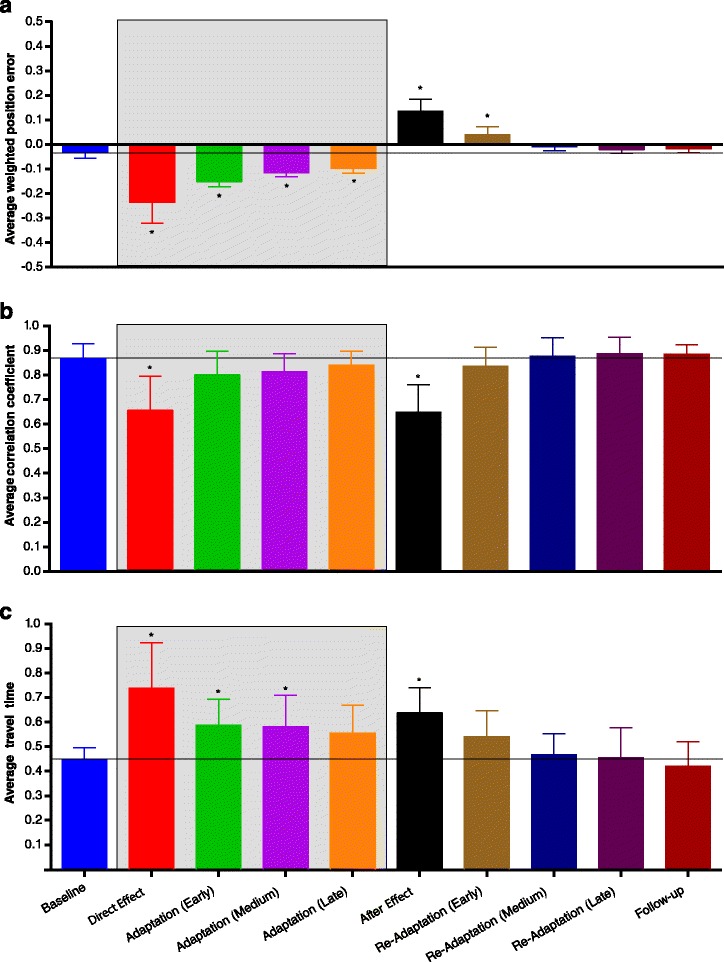
Kinematic metrics. Kinematic metrics for the different phases of the experiment averaged over all subjects and all steps: (**a**) average weighted position error in the *x* direction (*e*_*p*_), (**b**) average correlation coefficient between velocity profiles in a phase compared to the mean velocity profile in baseline (*c*_*p*_), (**c**) average travel time (*t*_*p*_). The error bars represent the inter-subject standard deviation, i.e. the standard deviation computed in each phase by using the metric of each subject as data set. The gray area indicates phases with exposure to the force field, asterisks indicate a significant difference with the baseline

Figure 2 shows the kinematic metrics and their standard deviations (inter-subject) during the main phases computed over all steps.

Statistical analyses indicated a significant interaction between direction and phase for the average weighted position error (interaction: *F*(9,81)=2.990, *p*=0.0040; direction: *F*(1,9)=7.548, *p*=0.0226; phase: *F*(9,81)=95.75, *p*<0.0001). However, for the comparisons considered (direct effect – baseline, direct effect – last adaptation, after effect – baseline, follow-up – baseline) we found no dependency on direction. Regarding the motion profiles, both the average correlation coefficient and the average travel time showed no interaction of the two factors (correlation: *F*(9,81)=0.564, *p*=0.8230; time: *F*(9,81)=0.597, *p*=0.7960), no effect of direction (correlation: *F*(1,9)=1.866, *p*=0.2051; time: *F*(1,9)=0.754, *p*=0.4078) but a significant effect of phase (correlation: *F*(9,81)=16.480, *p*<0.0001; time: *F*(9,81)=13.430, *p*<0.0001).

Pairwise post-hoc analyses indicated the presence of significant direct effects when the force field was first applied (error compared to baseline: *p*<0.0001) with a simultaneous change of the motion profile (significant reduction of correlation compared to baseline: *p*<0.0001) and an increase of the travel time (comparison with baseline: *p*<0.0001).

Following the direct effect, the subjects adapted to the alteration provided, reducing their position error and restoring more similar motion profiles. Indeed, all the metrics in the last adaptation phase differed significantly if compared with the direct effect (error: *p*<0.0001; correlation: *p*=0.0327; time: *p*=0.0003).

The performance showed also significant after effects when the perturbation was unexpectedly removed (error compared to baseline: *p*<0.0001). Velocity profiles changed both in shape (significant reduction of correlation compared to baseline: *p*<0.0001) and in time (significant increase of travel time with respect to baseline: *p*=0.0002).

Finally, the performance at follow-up was the same as the baseline (error: *p*=0.9956; correlation: *p*=0.9998; time: *p*=0.9992).

These results demonstrate the ability of healthy subjects to learn a viscous force field, as already known in literature [1], and suggest that all changes in muscular activity observed in this study can be associated with motor adaptation.

### Baseline motor modules

**Fig. 3:**
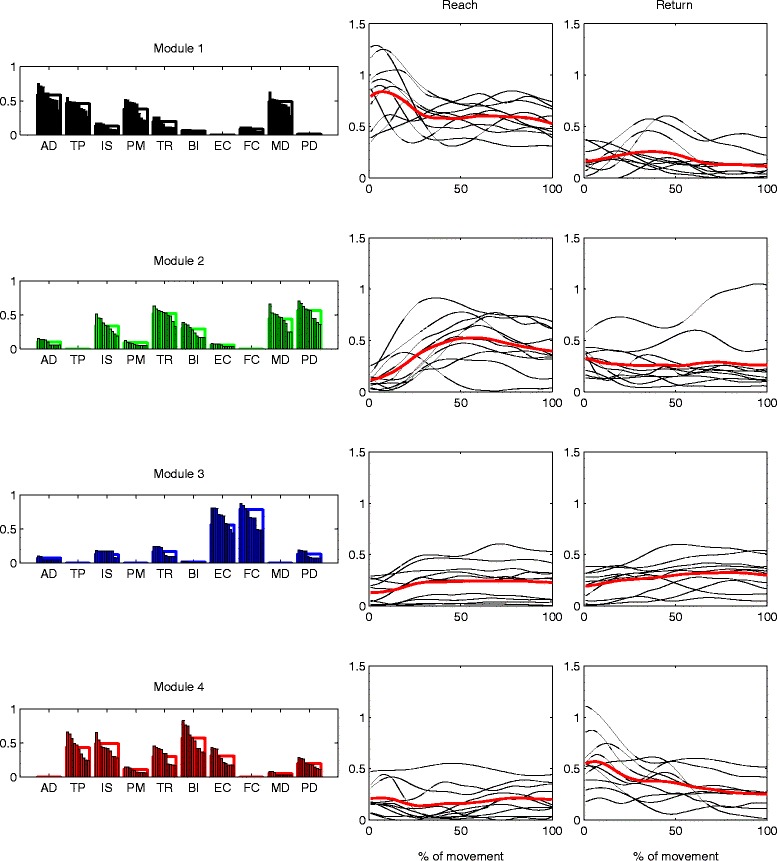
Motor modules and time-varying activation coefficients representing the baseline EMG data of all subjects. Four modules were sufficient to explain the baseline EMG data of all subjects with the desired accuracy, and were used also in all following phases. The average baseline activation coefficients of each subject are plotted next to each module (*black*), as well as the average activation coefficients over all subjects (*red*)

We found that four modules (*M*1 – *M*4) were sufficient to describe the EMG activity of all subjects during the baseline. The structure and the average time-varying activation of the motor modules extracted from the baseline are shown, respectively, in Figs. 3 and 5. Even though we allowed for individual variability of the muscle weights, the subject-specific versions of each module were very similar to each other and can be regarded as the same module, as the average between-subjects scalar product was higher than 0.9 for each module.

The activation appeared different between directions, phases, and modules (interaction among direction, phase and module: *F*(27,243)=2.5908, *p*<0.0001; direction: *F*(1,9)=18.1363, *p*=0.0021; phase: *F*(9,81)=5.2581, *p*<0.0001; module: *F*(3,27)=3.1765, *p*=0.0401). The post-hoc comparisons of the average activation between reach and return during baseline are reported in the first row of Table 1. The muscles that mainly participate in the activity of a module and the primary direction of action of each module are described below. To simplify the interpretation of each module, we considered only muscles with a weight higher than 0.35.

**Table 1:** Comparisons of the average activation between directions. Comparisons (*p* values) of the activation of the motor modules between reach and return for each phase (*w*_*rc,p,m*_– *w*_*rt,p,m*_). Up arrows indicate higher values in reach, down arrows higher values in return. Bolded values indicate statistical significance (*p*<0.05)

(*w*_*rc*_– *w*_*rt*_)_*p,m*_	*M*1	*M*2	*M*3	*M*4	*M*5	*M*7	*M*9	*M*6	*M*8	*M*10
**Baseline**	**0** **.** **0** **0** **0** **1** *↑*	0.1002	1.0000	**0** **.** **0** **0** **0** **1** *↓*	−	−	−	−	−	−
**Direct Effect**	**0** **.** **0** **0** **0** **1** *↑*	1.0000	**0** **.** **0** **0** **0** **1** *↓*	**0** **.** **0** **3** **3** **1** *↓*	0.8811	−	−	0.1787	−	−
**Adapt. (E.)**	**0** **.** **0** **0** **0** **1** *↑*	1.0000	0.0629	**0** **.** **0** **0** **2** **1** *↓*	−	0.1892	−	**0** **.** **0** **0** **3** **7** *↑*	−	−
**Adapt. (M.)**	**0** **.** **0** **0** **0** **1** *↑*	1.0000	1.0000	0.9993	−	0.1096	−	−	0.1533	−
**Adapt. (L.)**	**0** **.** **0** **0** **0** **1** *↑*	1.0000	1.0000	0.9593	−	**0** **.** **0** **1** **4** **2** *↓*	−	−	0.0863	−
**After Effect**	**0** **.** **0** **0** **0** **1** *↑*	0.9995	1.0000	0.3694	−	0.2899	−	−	0.9696	−
**Re-Adapt. (E.)**	**0** **.** **0** **0** **0** **1** *↑*	0.2665	1.0000	**0** **.** **0** **4** **2** **4** *↓*	−	−	0.9791	−	0.9938	−
**Re-Adapt. (M.)**	**0** **.** **0** **0** **0** **1** *↑*	0.4727	1.0000	0.0766	−	−	0.9903	−	1.0000	−
**Re-Adapt. (L.)**	**0** **.** **0** **0** **0** **1** *↑*	0.5550	1.0000	**0** **.** **0** **1** **5** **1** *↓*	−	−	0.9707	−	0.9996	−
**Follow-up**	**0** **.** **0** **0** **0** **1** *↑*	0.9053	0.3914	**0** **.** **0** **2** **8** **7** *↓*	−	−	0.9273	−	−	0.3280

- contained mainly AD, TP, PM, and MD and had a higher average activation during reach rather than during return (post test, *p*<0.0001). The participating muscles act primarily as shoulder flexors and abductors, as well as promoting humeral horizontal adduction and shoulder elevation, which is consistent with a higher activity during reach. Furthermore, we found that this module was significantly more active during the first part of reach (*p*=0.0245), indicating that it acted mainly during forward acceleration.
- represented mainly TR, MD, and PD muscles, which extend the elbow and the shoulder. We found no significant differences in average activation between reach and return (*p*=0.1002). However, we found that the module was more active during the last part of reach with respect to the first part (*p*=0.0076), which is consistent with the action of elbow extension as well as with an increased support of the extended limb provided by the deltoids.
- represented the co-activation of FC and EC, and was not significantly different between reach and return (*p*=1.0000). We interpret this module as a wrist stabilizer acting throughout the whole movement. During both reach and return, the module was more active during the last part of the movement (reach: *p*=0.0046, return: *p*=0.0094), suggesting that the higher accuracy required to hit the target was partly obtained by increasing wrist impedance.
- grouped primarily TP, IS, and BI and was more active during return (*p*<0.0001). This module could represent the concurrent elbow flexion, shoulder elevation and shoulder extension/external rotation required during the return movement. Additionally, the module was significantly more active during the first part of return (*p*=0.0013), indicating a higher contribution during backwards acceleration.

The analysis on the stability of the baseline modules showed that the second half of the baseline was reconstructed, by using the modules of the first half, with the same accuracy as the first half (VAF higher than 95 % for each subject), supporting the idea that any changes in motor modules found in the following phases are related to the adaptation process and not to a temporal instability of the baseline modules.

### Muscle coordination during exposure to the force field

As soon as the force perturbation was applied, we observed a significant change in the muscle coordination patterns. The modules extracted from the baseline were no longer able to fully describe the EMG activity of the following phases. Although we found significant phase-dependent changes in the activity level of the baseline modules, which we will describe in the next paragraphs, we did not observe significant changes in their timing profiles. Therefore their biomechanical interpretation given in the previous section remains still valid.

**Fig. 4:**
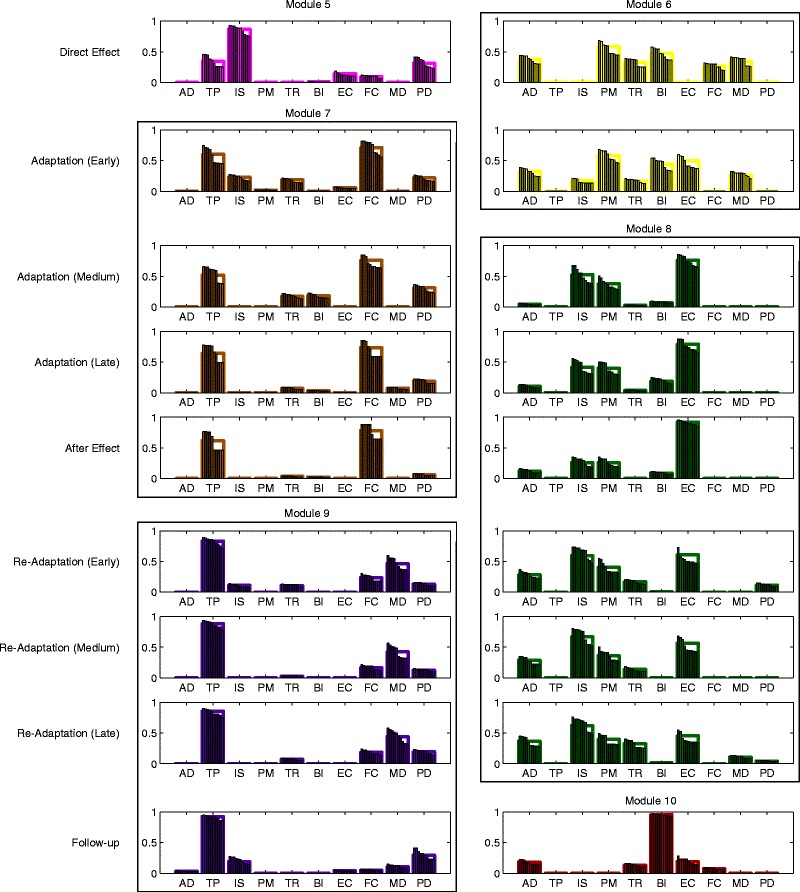
Complementary modules found during and after exposure to the perturbation. Each row shows the two additional complementary modules for the phases following baseline (from direct effect to follow-up). These modules are grouped into six clusters (*M*5−M10), representing additional modules which remain approximately unchanged for several subsequent phases

Starting from direct effect and in all following phases up to follow-up, two complementary modules were necessary to describe the muscle activity with the desired accuracy. The total number of modules was therefore six per phase. We observed a high similarity between complementary modules extracted from different phases. The k-means cluster analysis revealed that the 18 complementary modules could be grouped into 6 clusters, whose centroids we will call respectively *M*5−*M*10 (Fig. 4).

The two complementary modules describing changes in muscle activation right after exposure to the force field were of temporary nature, and quickly evolved into more stable modules spanning multiple phases.

- grouped mainly TP and IS muscles and was found solely in the direct effect phase. Its average activity was not significantly different between reach and return (pairwise t-test: *p*=0.8811).
- consisted mainly of AD, PM and BI. From the analysis of the average activation of *M*6, we found no significant interaction between direction and phase (*F*(1,9)=3.0870, *p*=0.1128) with significant effects of direction (*F*(1,9)=10.2800, *p*=0.0107) and phase (*F*(1,9)=6.753, *p*=0.0288). Post-hoc comparisons between reach and return revealed that, in early adaptation only, the module was significantly more active during reach movements (*p*=0.0037). Also, the average activation resulted higher in early adaptation when compared to direct effect (reach: *p*=0.0266).

Together with the change in muscle coordination patterns, we also observed a shift in the activation of some baseline modules. The average activity during direct effect of *M*1, *M*2 and *M*3 differed from that shown at baseline. Specifically, post-hoc comparisons revealed lower values for *M*1 and *M*2 (reach: *p*=0.0325; *p*=0.0093), and higher values for *M*3 (return: *p*=0.0003). This difference was maintained by *M*1 in early adaptation (*p*<0.0001).

With the early adaptation phase participants started to develop more stable motor modules, which maintained their structure over several phases:

- substituted *M*5 during early adaptation and was found until the after effect phase. Similarly to *M*5 it showed a high participation of TP, while the weight of IS was significantly reduced and the contribution of FC was increased. Its average activation showed interaction between direction and phase (interaction: *F*(3,27)=5.3440, *p*=0.0051; direction: *F*(1,9)=1.0510, *p*=0.3321; phase: *F*(3,27)=1.9710, *p*=0.1421). Post-hoc tests revealed no significant difference between reach and return (*p*>0.1096) before the late adaptation phase, in which it significantly increased during returns (*p*=0.0142). Also, the average activation tended to reduce when the adaptation to the force field was improved (comparison between early and late adaptation in reach: *p*=0.0023).
- replaced *M*6 during medium adaptation, and lasted to late re-adaptation. It grouped mainly IS and PM and EC. No differences were found in the average activation between directions (interaction: *F*(5,45)=1.8980, *p*=0.1135; direction: *F*(1,9)=0.5248, *p*=0.4872; phase: *F*(5,45)=22.9900, *p*<0.0001).

During medium and late adaptation the activity of *M*1, *M*2, and *M*3 returned to baseline levels, while *M*4 lost its asymmetry between reach and return movements (*p*>0.9593) due to a significant decrease in activity during return compared to baseline (medium adaptation – baseline: *p*=0.0400; late adaptation – baseline: *p*=0.0123).

### Muscle coordination after exposure to the force field

After removal of the force field, the structure and activity of all motor modules remained mostly unchanged and we found no significant difference in their activity between late adaptation and after-effect (*p*>0.1632). The only difference during after-effect was found in the activity of *M*7, which was, on average, more similar between reach and return movements (*p*=0.2899).

These findings show that participants tended to maintain the same muscle coordination patterns used during adaptation to the force field, even though the force had been abruptly removed.

After this initial trend to maintain the same coordination patterns, we observed a slight change in the structure of one of the complementary modules during early re-adaptation:

- lasted until late re-adaptation with a balanced activity between reach and return (*p*>0.9938). However, its average activation significantly decreased during re-adaptation (comparison between after effect and late re-adaptation: *p*=0.0209 in reach; *p*=0.0008 in return).
- replaced *M*7 from early re-adaptation to follow-up. While the overall structure of the module was very similar to *M*7, with a high participation of TP, the weight of FC was drastically reduced and MD started to significantly contribute to the module. Its average activation was balanced between reach and return, and similar between phases (interaction: *F*(3,27)=0.3648, *p*=0.7789; direction: *F*(1,9)=0.0805, *p*=0.7831; phase: *F*(3,27)=0.5164, *p*=0.6745).

During re-adaptation, *M*1, *M*2, and *M*3 maintained their activation patterns in the two movement directions (*M*1: *p*<0.0001; *M*2: *p*>0.2665; *M*3: *p*=1.0000), whereas *M*4 returned to be more activated during return movements with respect to reaches (early re-adaptation: *p*=0.0424; late re-adaptation: *p*=0.0424), except in medium re-adaptation (*p*=0.0766).

The effects of motor adaptation were observed also in the follow-up phase, as we still found six modules. However, after the five-minute break between late re-adaptation and follow-up, one of the previous complementary modules was substituted by a new one:

- was found also at follow-up and showed the same activation pattern, with no significant difference between reach and return and between re-adaptation and follow-up.
- replaced *M*8 in this phase and was constituted mainly by BI. Its average activity was not significantly different between directions (pairwise t-test: *p*=0.3280).

The activities of all the baseline modules showed a comparable behavior to the baseline, both between directions (*M*1: *p*<0.0001; *M*2: *p*=0.9053; *M*3: *p*=0.3914; *M*4: *p*=0.0287) and between phases (*p*>0.4540 in reach and return).

When analyzing the timing of the complementary modules with respect to the end point velocity profile, we observed a high variability from subject to subject. We were therefore unable to detect significant statistical differences both between phases and directions.

A qualitative analysis on the average values across subjects (Table 2) suggests that the timing of the complementary modules is slightly delayed during direct effect and then becomes more synchronous as the adaptation to the perturbation progresses (eventually even anticipating the perturbation in the case of *M*7). Following the after effect phase, the timing becomes more variable, which is consistent with the absence of the external perturbation. However, these results need to be interpreted with caution.

**Table 2:** Average synchronization between velocity and module activation. Difference in time between the peak in module activation and the velocity peak, averaged across subjects and across directions, and normalized by movement time. Positive values indicate that the peak of module activity is delayed with respect to the velocity peak

$\overline {\Delta } t_{p,m}$	*M*5	*M*7	*M*9	*M*6	*M*8	*M*10
***Baseline***	−	−	−	−	−	−
***Direct Effect***	16.4 *%*	−	−	11.0 *%*	−	−
***Adapt. (E.)***	−	2.0 *%*	−	13.6 *%*	−	−
***Adapt. (M.)***	−	−1.0 *%*	−	−	6.0 *%*	−
***Adapt. (L.)***	−	−9.2 *%*	−	−	3.3 *%*	−
***After Effect***	−	−9.5 *%*	−	−	11.4 *%*	−
***Re-Adapt. (E.)***	−	−	−11.4*%*	−	15.7 *%*	−
***Re-Adapt. (M.)***	−	−	0.0*%*	−	7.1 *%*	−
***Re-Adapt. (L.)***	−	−	−3.3*%*	−	8.7 *%*	−
***Follow-up***	−	−	−22.6 *%*	−	−	−1.3 *%*

## Discussion

In this study, we investigated whether, during reach and return movements, the adaptation to a dynamic perturbation induced changes in the muscle coordination patterns of healthy subjects. Muscle coordination of the exercised upper limb was described using motor modules as analytical tool, which allowed us to decompose the activity of the 10 recorded muscles into a limited set of functional building blocks (modules).

We identified four modules describing the muscle activity during the unperturbed baseline condition. Module *M*1 was primarily active during forward movements (reach) and could be associated with shoulder flexion and abduction. Modules *M*3 and *M*4 acted preferentially in the opposite direction (return), stabilizing the wrist (*M*3) and promoting elbow flexion and shoulder extension, in addition to wrist extension (*M*4). The activity of *M*2 could not be distinguished between reach and return movements, which could either indicate that it was similarly active in the two directions, or that there was a high variability between subjects. From a biomechanical point of view, the muscles participating in *M*2 could contribute both in decelerating the arm during the last part of reach, as well as in accelerating the arm during the first part of return. This could partially justify the similarity in average activity between the two directions.

The baseline modules explained also a significant part of the EMG signals of all subsequent phases of the experiment, i.e. the exposure to a dynamic perturbation and the re-adaptation to an unperturbed environment. However, they were not sufficient to completely describe muscle activation in such phases with the required accuracy.

**Fig. 5:**
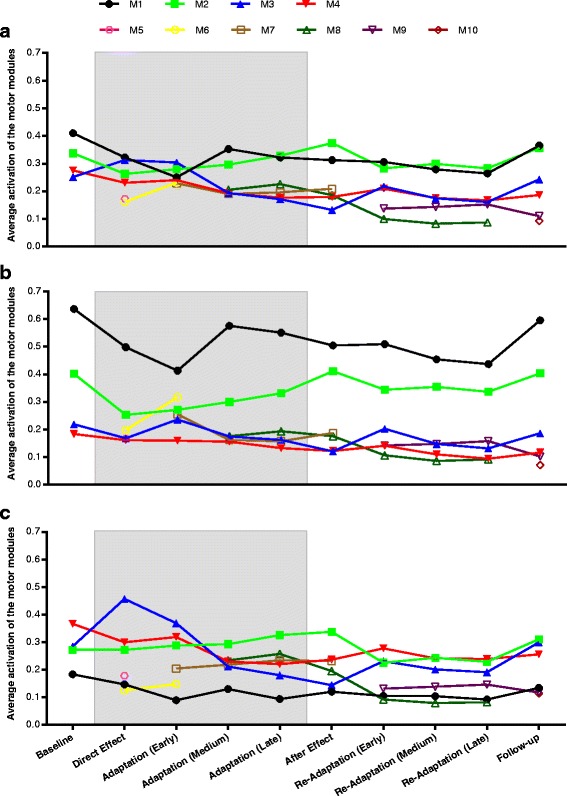
Average activation of the motor modules. Activation of each motor module in different phases of the exercise averaged over all subjects: (**a**) all steps (*w*_*p,m*_), (**b**) reaches only (*w*_*rc,p,m*_), (**c**) returns only (*w*_*rt,p,m*_). The gray area indicates phases with exposure to the force field

We found that the evolution of muscle coordination patterns during and after exposure to the viscous force field took place in two stages. Right after the application of the force field (direct effect) and partly during early adaptation, subjects counteracted the perturbation by adjusting their muscle coordination to the new condition. Our analysis detected these changes in two ways: a change in activation of the baseline modules and the use of two additional temporary modules (*M*5 and *M*6) summarizing previously unused coordination strategies. We found a significant decrease in the activity of modules *M*1 and *M*2, and an increased activity of *M*3 (Fig. 5), indicating that subjects tended to co-contract EC and FC to stiffen the wrist joint. On the other hand, the decreased activity of *M*1 and *M*2 was partly counterbalanced by modules *M*5 and *M*6. Such modules grouped mainly shoulder and elbow muscles, promoting shoulder elevation and extension (*M*5) and shoulder flexion, humeral horizontal adduction and elbow flexion (*M*6). Interestingly, in this phase of the experiment subjects did not adopt modules which could directly counteract the external force in the mediolateral direction: instead of adopting modules that would primarily act either in the medial or in the lateral direction, subjects initially reacted to the perturbation by increasing joint stiffness. This strategy, based on co-contracting muscle groups, requires a greater effort than counteracting the external force by selectively activating muscle groups [34]. However, it allowed subjects to contain kinematic errors in this stage, while they had not yet adapted to the perturbation and the trajectory errors resulted large (Fig. 2).

In a second stage, subjects were able to adapt more effectively to the perturbation, as evidenced by smaller kinematic errors. We found complementary modules which were substantially different from those seen in the first stage, representing a new approach to the perturbation. Notably, the action of FC and EC began to be separated: *M*7, which replaced *M*5, showed a significantly higher weight of FC; *M*6 changed by increasing the weight of EC. This indicates that subjects started to use muscle activation patterns which could act against the external force in both the medial and the lateral direction. This behavior was consistent with the literature, in which agonist-antagonist muscle co-contraction was found to increase on exposure to the novel dynamics and then decrease as learning progressed [35–37], as a minimization of kinematic error and effort [34, 38].

This transition became more evident in medium adaptation, when *M*6 was replaced by *M*8. This new module contributed mainly in extending the wrist and externally rotating the humerus, thereby directly counteracting a force acting in the medial direction. The contribution of PM in *M*8 partially contrasted the action of IS, and probably acted to stabilize the glenohumeral joint. On the other hand, *M*7 contributed mainly in flexing the wrist and elevating the shoulder, counteracting forces in the lateral direction. Due to the limitations of surface EMG we were not able to collect activation data of shoulder internal rotators, which are likely to participate in the activity of *M*7.

The slow response of muscle coordination to changes in the environment, as seen in the direct effect phase, was also observed during after effect: subjects tended to maintain the same muscle coordination patterns used during adaptation to the force field, even though the force had been removed. In the following phase (early re-adaptation), *M*7 was replaced by *M*9. Module 9 was characterized by a lower weight of FC, which was no longer needed after the removal of the external force. At the same time, and consistently with the decrease of the weight of FC in *M*9, also the weight of EC in *M*8 started to decrease. It is interesting to note that subjects maintained a modular structure that was strikingly similar to that used during the perturbed condition, indicating that motor adaptation had influenced the muscle coordination patterns. This effect was also partly observed during follow-up, as *M*9 was still present. In this last phase, *M*8 was substituted by *M*10, showing that part of the adopted coordination strategies had been lost over a relatively short time. However, even though the structure of all modules was not completely maintained, the total number of motor modules did not return to the baseline level, which is evidence for a higher complexity of muscle activation patterns achieved after the experiment.

One limitation of this study is the specificity of the extracted modules to the performed task. As shown by other studies [39, 40], a generalized set of motor modules used by a subject can only be obtained by extracting them from a wide variety of tasks. In this study, we chose to extract motor modules from a single task, thereby obtaining modules that are representative only of the muscle coordination for that specific task. This allowed us to detect changes in muscle coordination for the tested task, but leaves an open question regarding the mechanism behind these changes (i.e. whether they originated from a re-weighting of the generalized set of modules or from the development of new modules). Clearly, this will need to be addressed in the future.

A second limitation is the high variability in the module activation coefficients found across subjects, which limited the analyses we could perform on the module’s timing. In our future experiments we will need to modify the experimental setup in order to elicit higher end point forces and more consistent muscle activation timing.

## Conclusion

The question whether muscle coordination can be changed by interacting with a haptic device is of great importance for the field of robot aided neurorehabilitation. Robots can be adjunctive tools that increase the intensity of rehabilitation therapies with the aim of improving motor function and activities of daily living of patients [41–43]. Robotic therapy proved itself to be at least as effective as traditional therapy [44, 45], also when used in partial substitution of conventional rehabilitation treatment [46]. However, the effects of robotic therapy on muscle coordination are still not well understood. This aspect of robotic rehabilitation needs to be further investigated, especially because there is evidence that muscle coordination patterns are altered after neurologic injury such as stroke [28, 47–50].

Our study showed that alterations in muscle coordination during and after exposure to a force field could be captured by extracting complementary motor modules. These complementary modules summarize coordination strategies that were not used before the perturbation, and which mainly recruit muscles involved in counteracting the force produced by the device. These results may serve as an example of how muscle coordination patterns can be analyzed during and after robotic rehabilitation, and may be considered a first step towards targeting rehabilitation to address specific muscle coordination deficits. Clearly, the effects seen on healthy individuals need to be confirmed on stroke patients. Secondly, future studies should address whether specific coordination patterns can be targeted by adjusting the force fields generated by the robot, and to what extent they need to be tailored to the deficit of each patient and/or to the recovery phase in which the robotic treatment is delivered [43].
